# Use of moulded hearing protectors by child care workers - an interventional pilot study

**DOI:** 10.1186/s12995-016-0138-1

**Published:** 2016-11-08

**Authors:** Peter Koch, Johanna Stranzinger, Jan Felix Kersten, Albert Nienhaus

**Affiliations:** 1 Centre of Excellence for Epidemiology and Health Services Research for Healthcare Professionals (CVcare), University Medical Centre Hamburg-Eppendorf, Martinistraße 52, Hamburg, 20246 Germany; 2 Health Protection Division (FBG), Institution for Statutory Accident Insurance and Prevention in the Health and Welfare Services (BGW), Pappelallee 33, Hamburg, 22089 Germany

**Keywords:** Moulded hearing protectors, Child care worker, Burnout, Personal hearing protector, Noise exposure

## Abstract

**Background:**

Employees of a multi-site institution for children and adolescents started to wear moulded hearing protectors (MHPs) during working hours, as they were suffering from a high level of noise exposure. It was agreed with the institutional physician and the German Institution for Statutory Accident Insurance and Prevention in the Health and Welfare Services (BGW) that this presented an opportunity to perform a scientific study to investigate potential beneficial effects on risk of burnout and subjective noise exposure at work when child care workers wear MHPs.

**Methods:**

This was an intervention study which compared the initial values with those after a follow-up of 12 months. All teaching child care workers employed by the multi-site institution were offered the opportunity to take part. Forty-five (45) employees in 16 institutions participated. The subjects were provided with personally adapted MHPs and documented the periods of wear in a diary. At the start and end of the intervention, the subjects had to answer a questionnaire related to subjective noise exposure and burnout risk. In parallel, employees were surveyed who had not taken part in the intervention.

**Results:**

Thirty-three (33) subjects took part in the follow-up after 12 months (follow-up rate 73 %). The median period of wear of MHPs was 34.6 h. During the period of observation, the mean subjective noise exposure increased by 2.7 %, and mean burnout risk by 2.5 scale points (baseline: 55.2, follow-up 57.7). Neither difference was statistically significant. 67 % of the participants reported that they were still capable of fulfilling their teaching duties when wearing the MHPs. In the reference group without the intervention, the increase in burnout risk was 3.9 points, which was even less favourable (baseline: 50.6, follow-up: 54.5).

**Conclusions:**

Within the working environment of the child care workers, wearing MHPs did not reduce subjective noise exposure or burnout risk; the satisfaction of the study subjects with wearing MHPs decreased over time. There were however signs that the level of stress increased over time and that this might have been alleviated in the intervention group by wearing MHPs.

## Background

Child care workers in day care centres or other institutions for children and adolescents are continuously exposed to noise from children throughout the working day. Objective measurements have found that the peak sound pressure in these institutions is greater than 85 dB(A) [1–5], which confirms the employees’ subjective impression [1, 6, 7]. This noise is mostly caused by the children’s voices and their playing [4]. This may be exacerbated by poor conditions, for example, if the ratio of children to child care workers is high, or if the structure of the building is unsuitable. Studies have shown that staff report fewer health problems when they work in institutions with closed rooms than in those with large half open or open rooms [8]. In this setting, symptoms associated with noise include headache, exhaustion, burnout, stress, voice problems, hearing difficulties and tinnitus [4, 7–12]. In environments with continuous background noise, small children are even at risk of disturbances in speech development [13]. A study with preschool children has shown that children who are exposed to a higher level of noise are more likely to have problems in learning to read [14].

The most usual interventions to reduce noise in child day care centres are technical or organisational. These include, for example, increased noise insulation, the selection of special toys and furniture, using noise warning lights, noise education for children or designating withdrawal rooms for the staff. However, studies on these interventions show that they have little efficacy on subjective noise exposure suffered by employees [11, 15].

In the industrial working environment, the efforts to reduce hearing damage are not only technical or organisational, but may be individualised. In Germany, a personal hearing protector is required if noise exposure exceeds 80 dB(A); this is based on EU Directive 2003/10/EU [16] and was incorporated in the Noise and Vibration Occupational Safety Health Ordinance [17]. In workplaces with technical noise, many employees wear a capsule hearing protector, e.g. soldiers, farmers and industrial workers [18–22]; moulded hearing protectors (MHPs) are often worn by professional musicians. In comparison to capsule hearing protectors, MHPs with adjustable otoplasty filter systems have the advantage that speech is still comprehensible with the hearing protector. For example, in occupational medicine, they are selectively used for teachers and child care workers with hyperacusis. We are unaware of any scientific studies on the use of MHPs in child care workers.

In this context, the following questions were examined in our study:

- Does the use of MHPs reduce subjective noise exposure among child care workers?
- Does the use of MHPs reduce risk of burnout among child care workers?
- Do child care workers find the use of MHPs to be comfortable and feasible?
- What are the acoustics (reverberation time) of critical rooms that were identified by an experienced acoustician and are there associations between acoustic properties of the rooms and subjective noise exposure and risk of burnout respectively?

## Methods

**Fig. 1:**
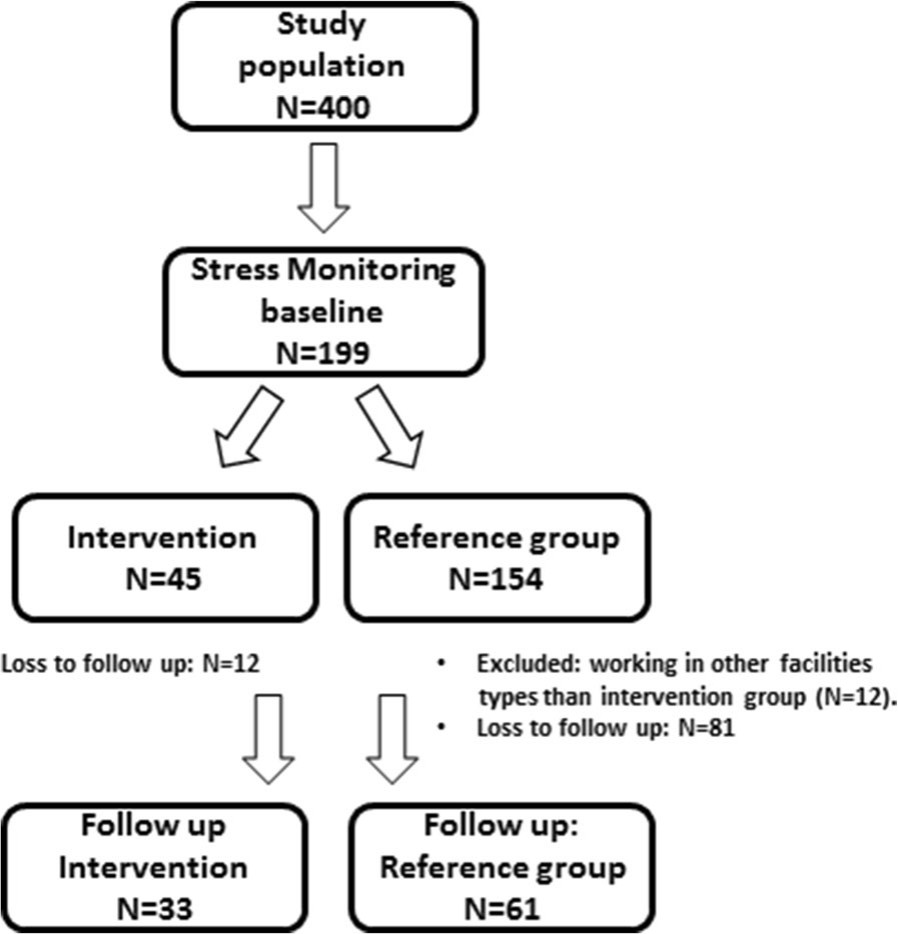
Inclusion Flowchart

As part of stress monitoring for child care workers in a multi-site institution for children and adolescents [7], parallel groups of subjects were recruited for an interventional study on reduction in risk of burnout and subjective noise exposure by MHPs. The participation in the study was offered all 400 child care workers in 26 different facilities (Fig. 1). The multi-site institution bears the responsibility for caring for children in three different types of institutions: 1) day care centres for children aged up to 6 years, 2) school partnerships for school children and 3) facilities to support children and adolescents, e.g. sheltered housing groups. A total of 45 subjects were recruited for the intervention study and also participated in stress monitoring at the same time. The efficacy and practicability of the MHPs were assessed by comparing the initial measurements with those after one year of observation. Post hoc, to examine the results from another perspective, a comparator group was made up of subjects who only took part in stress monitoring. This comparison was carried out so that it would be possible to assess whether the stress potential in the institutions had remained constant or changed over time. As this is not a classical control group, it will be referred to below as the “reference group”. 12 subjects working in facilities to support children and adolescents have been excluded from the reference group as in the intervention group no one was working in this type of institution. Overall the reference group comprised 61 subjects. There were no statistically significant differences in demographic characteristics between the intervention and reference group. The inclusion criteria for participation in the intervention were as follows: a) participation in stress monitoring, b) employment in teaching, c) intended employment for at least another year, d) minimum age of 18 years. In order to avoid windfall effects, each subject had to contribute 20 Euros towards their personal hearing protector. The subjects came from 16 different institutions; 11 of these were kindergartens and 5 were school partnerships, in which children from fulltime schools were looked after during teaching breaks.

The pseudoanonymous questionnaire was agreed with the multi-site institution’s data protector officer. All study documents including the study protocol were reviewed and approved by the Ethics Committee of the Hamburg Medical Association (Reference Number: PV4792). Each subject provided a signed declaration of consent.

### Intervention

After taking the imprints of the outer ear, the audiologist presented the 45 subjects with a workshop on dealing with MHPs and on the issue of noise. A central recommendation of the workshop was to use the MHPs punctually in situations with high noise exposure (e.g. during lunch). The intervention started in October 2014 (T0) and the subjects were then sent their individually prepared MHPs (Variphone “MEP-2G” Hearing Protector) by post and completed the form on stress monitoring. At the same time, they were given a diary, in which they had to document the times they used the personal hearing protector each day. After five months (T1), the subjects had the opportunity of visiting the audiologist again, in order to modify the fit and the filter strength of their MHPs. At this time, the user satisfaction was recorded with a short questionnaire. After a total of 12 months (T2) follow-up, questionnaires were distributed again on stress monitoring and user satisfaction.

### Questionnaire

The questionnaire on stress monitoring collected data on the following factors that could potentially influence the effect of the intervention: three standardized questionnaires components (effort reward imbalance [23], physical stress [24], work-related stress and resources from the Short Questionnaire on Work Analysis (KFZA) [25]). The effort reward imbalance (ERI) questionnaire consists of three dimensions, *effort, reward* and *Overcommitment.* The ERI questionnaire assesses the psychosocial situation of the worker. In studies internal consistencies were satisfactory and varied between 0.70 and 0.91 (Cronbach’s alpha) [26, 27]. The KFZA questionnaire comprises different work-related strains and resources. The following scales have been chosen for our questionnaire: *control, variety, entirety, cooperation, qualitative workload, quantitative workload, work disruption and information.* For qualitative workload and work disruption internal consistencies were 0.40 and 0.44 (Cronbachs alpha). For all other scales Cronbachs alpha was between 0.51 and 0.71 [25]. For the questionnaire on physical stress, no study on reliability was available. *Subjective noise exposure* was determined from a cumulative score (range 13–65 points) out of 13 self-developed rating items. Questions such as “There are rooms where I hear particularly poorly” or “This level of noise bothers me” were answered on a 5-point scale, ranging from *strongly agree* to *strongly disagree.* Regarding everyday working life situations for child care workers nine self-developed questions have been added to the questionnaire. To assess situations that might include a stress potential, questions such as “Breaks and possibilities for recovery are missing” or “There are conflicts with parents” were answered on a 4-point scale ranging from *strongly agree* to *strongly disagree.*

Two outcome variables were examined: burnout risk and subjective noise exposure; the latter may be a factor that influences burnout risk. Burnout risk was assessed on the basis of the subscale *personal burnout* of the Copenhagen Burnout Inventory [28]. In the German version of the Copenhagen Psychosocial Questionnaire (COPSOQ) questionnaire the subscale *personal burnout* shows a Cronbach’s alpha of 0.91 [29]. The self-developed questionnaire on user satisfaction collected information on wearing comfort, acoustic perception and the reasonableness of wearing MHPs at work. The answers were rated on the basis of a 5-point rating scale and were then dichotomised due to presentational reasons (yes = absolutely or predominantly true; no = partially correct, predominantly incorrect, absolutely incorrect).

### Acoustic measurements

In order to have not only subjective but also objective data on room acoustics, reverberation times were measured in the various institutions during the observation period. The measurements were in the frequency range of 125–4000 Hz and were carried out in rooms with different functions - playrooms, group rooms, classrooms, movement rooms, flights of stairs and restaurants for children. The results were compared with the target values in DIN Norm 18041 on acoustic properties in rooms of small or intermediate size [30]. On the basis of the reverberation times and building properties, an expert in room acoustics performed a final evaluation of the selected rooms. Between 1 and 4 rooms were evaluated for each institution, except one institution of school partnership without any measurement.

### Power estimation

During the run-up, it was assumed that about 50 child care workers would be interested in taking part in the study.

On the basis of this number of evaluable cases, it was postulated that the outcome parameter of mean subjective noise exposure would exhibit the necessary normal distribution. With an assumed difference in the means of 2 points before and after the intervention, with a standard deviation of 5 points and an alpha of 5 %, the power of 80 % was calculated.

### Statistical analysis

The difference in the means for the comparison before and after the intervention was calculated with the *t* test for dependent samples, for not normally distributed and dependent data the Wilcoxon signed-rank test was used. Group comparisons were based on the *t* test for independent samples. Analysis of variance (ANOVA) and multivariate linear regression were used to test potential factors for the differences in the means of the outcome variables - subjective noise exposure and burnout risk (T2-T0), all analyses were performed on the individual level. The following factors were measured at T0 and were considered as factors potentially influencing the outcome variable *subjective noise exposure*: *work-related resources, everyday situations at work, effort reward imbalance, overcommitment, physical stress, weekly working hours, type of institution, physical exercise,* and time of *wearing MHPs.* To characterize the cohort demographic variables like age, gender and BMI were assessed too. For the analysis of the outcome variable *burnout risk*, the additional variable *subjective noise exposure* was investigated as a covariate. Independent variables with a level of significance of *p* > 0.2 in the bivariate analysis were excluded from the analyses. In addition, a drop-out analysis with all variables was performed by logistic regression. If there were statistically significant predictors, these were included as covariates in the linear model. Level of significance was set to two-tailed *p* < 0.05. Statistical analysis was performed with the program SPSS Version 22.

## Results

After the follow-up period of 12 months, questionnaires had been received from 33 subjects (follow-up rate: 73 %). Table 1 describes the demographic characteristics of the cohort at time T0. Most subjects were women (91 %). The largest age group consisted of subjects between 40 and 50 years old (38 %). 40 % had a BMI of over 25 and 56 % were regularly engaged in sporting activities. At that point in time, 40 % were working fulltime; most worked in child day care centres.

**Table 1:** Demographic characteristics of the subjects

Variable	N	Percentage
Gender		
Women	41	91.1 %
Men	4	8.9 %
Age in years		
18 to <30	9	20 %
30 to <40	12	26.7 %
40 to <50	17	37.8 %
50+	7	15.6 %
Nationality		
German	43	95.6 %
Other	2	4.4 %
BMI		
< 25	25	55.6 %
≥ 25	18	40 %
Missing	2	4.4 %
Physical exercise		
Regularly	25	55.6 %
None	20	44.4 %
Weekly working hours		
Fulltime	18	40 %
Part time	27	60 %
Institution		
Child day care centre	36	80 %
School partnership	9	20 %
Support for children and adolescents	0	0 %
Total	45	100 %

**Fig. 2:**
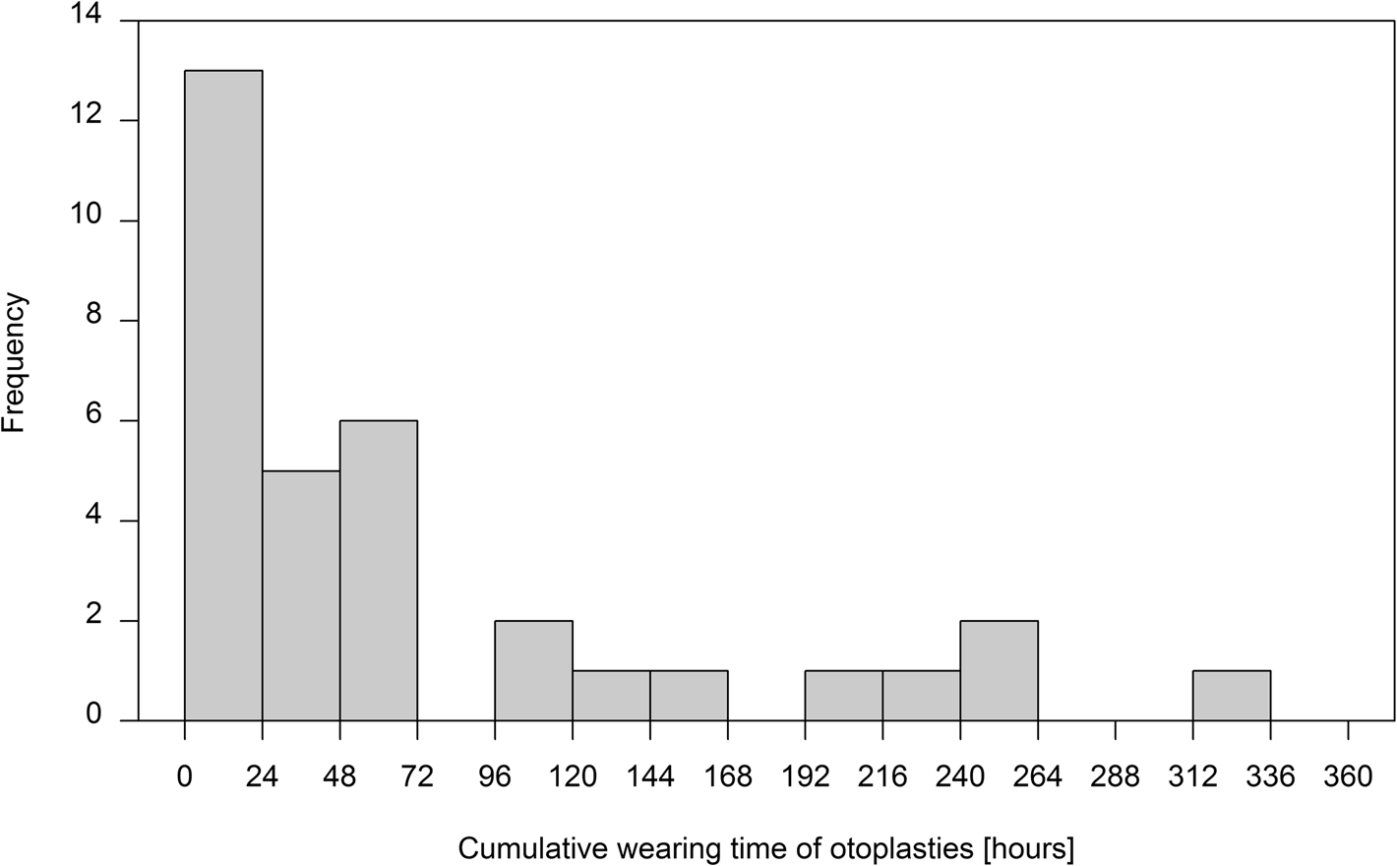
Cumulative wearing time of MHPs (*N* = 33)

Figure 2 shows the cumulative wearing time in hours; there were no entries for 6 persons in the diaries. The median of the cumulative wearing time was 34.6 h (range: 0–326). The median cumulative number of days on which the MHPs were worn was 25 days (range 0–174 days).

There were no statistically significant differences between the intervention and reference group with respect to the demographic and all other independent variables e.g. effort reward imbalance. Among the outcome variables, only the difference of the baseline values in subjective noise exposure was statistically significant (*p* = 0.004). Table 2 shows the changes in the outcome variables for the two groups. In the intervention group, mean subjective noise exposure increased from 44.5 to 45.7 points over time (reference group: from 38.1 to 39.7). The difference in subjective noise exposure was greater in the reference group than in the intervention group (Δ = 1.6 vs. 1.2). The difference in the development of subjective noise exposure between the two groups was not statistically significant. In the intervention group, the burnout risk increased from 55.2 to 57.7 (Δ = 2.5) points. The increase in the reference group was greater - from 50.6 to 54.5 points (Δ = 3.9). None of the differences between the groups was statistically significant.

**Table 2:** Description of the outcome variables in the intervention and reference groups

	Intervention group (*N* = 33)*			Reference group (*N* = 61)*		
	T0	T2	*p*	T0	T2	*p*
Subjective Noise Exposure	44.5 (8.8)	45.7 (7.9)	0.30	38.1 (10.3)	39.7 (10.5)	0.08
Δ Subjective Noise Exposure T2-T0	1.2 (7.4)		NA	1.6 (6.8)		0.80
Burnout Risk Scale	55.2 (19.4)	57.7 (17.7)	0.40	50.6 (19.7)	54.5 (22.1)	0.05
Δ Burnout Risk Scale T2-T0	2.5 (18.3)		NA	3.9 (15.3)		0.70
Burnout Risk Scale ≥ 50	66.7 %	72.7 %	0.62	54.1 %	62.3 %	0.22

*given values are means (standard deviations), rates and *p*-values

Table 3 shows the means of the outcome variables subjective noise exposure and burnout risk for the institutions with or without a recommendation for improvements in room acoustics. For subjective noise exposure at time T0, there was a small difference between the two groups (45.8 vs. 42.2, Δ = 3.6). The difference was slightly greater on follow-up (47.7 vs. 42.8, Δ = 4.9). After 12 months, the difference (Δ T2-T0) for participants from institutions with recommendation was greater than for the other group (Δ = 1.9 vs. 0.7) but statistically not significant. The differences in burnout risk were greater: at time point T0 (59.2 vs. 48.3), the difference was 10.9 and, at time point T2 (60.8 vs. 52.1), the difference was 8.7 points on the burnout scale. At time point T2 the differences in burnout risk (Δ T2-T0) for workers from institutions without a recommendation was more than double high than for the comparison group (Δ = 3.8 vs. 1.6) However, none of these differences was statistically significant.

**Table 3:** Outcome variables for subjects in institutions with or without a recommendation for improvement in room acoustics

	Acoustic improvements recommended? Yes (*N* = 20), No (*N* = 12)					
	Subjective Noise Exposure*			Burnout Risk Scale*		
	Yes	No	*p*	Yes	No	*p*
T0	45.8 (9.3)	42.2 (8.2)	0.26	59.2 (19.9)	48.3 (19.8)	0.13
T2	47.7 (7.2)	42.8 (8.8)	0.12	60.8 (19.6)	52.1 (14.1)	0.15
Δ T2-T0	1.9 (8.2)	0.7 (7.6)	0.67	1.6 (20.7)	3.8 (15.4)	0.75

*given values are means (standard deviations) and *p*-values

Multivariate analysis demonstrated that the feature *Breaks and possibilities for recovery are missing* had a significant effect on subjective noise exposure (B = 5.7, *p* = 0.013) (Table 4). According to this, the effect on subjective noise exposure for this subgroup became even less favourable over time. All other potential factors exhibited no significant effects and were therefore excluded from the model. No statistically significant effects on burnout were identified.

**Table 4:** Results of multivariate linear regression (*N* = 32), adjusted for subjective noise exposure at time point T0

Dependent variable: Difference in subjective noise exposure T2-T0			
Factor	Effect	95 % CI	*P*
Breaks and possibilities for recovery are missing (1 = yes, 0 no)	5.7	1.29–10.13	0.013
	*R* ^2^ = 0.42		

**Fig. 3:**
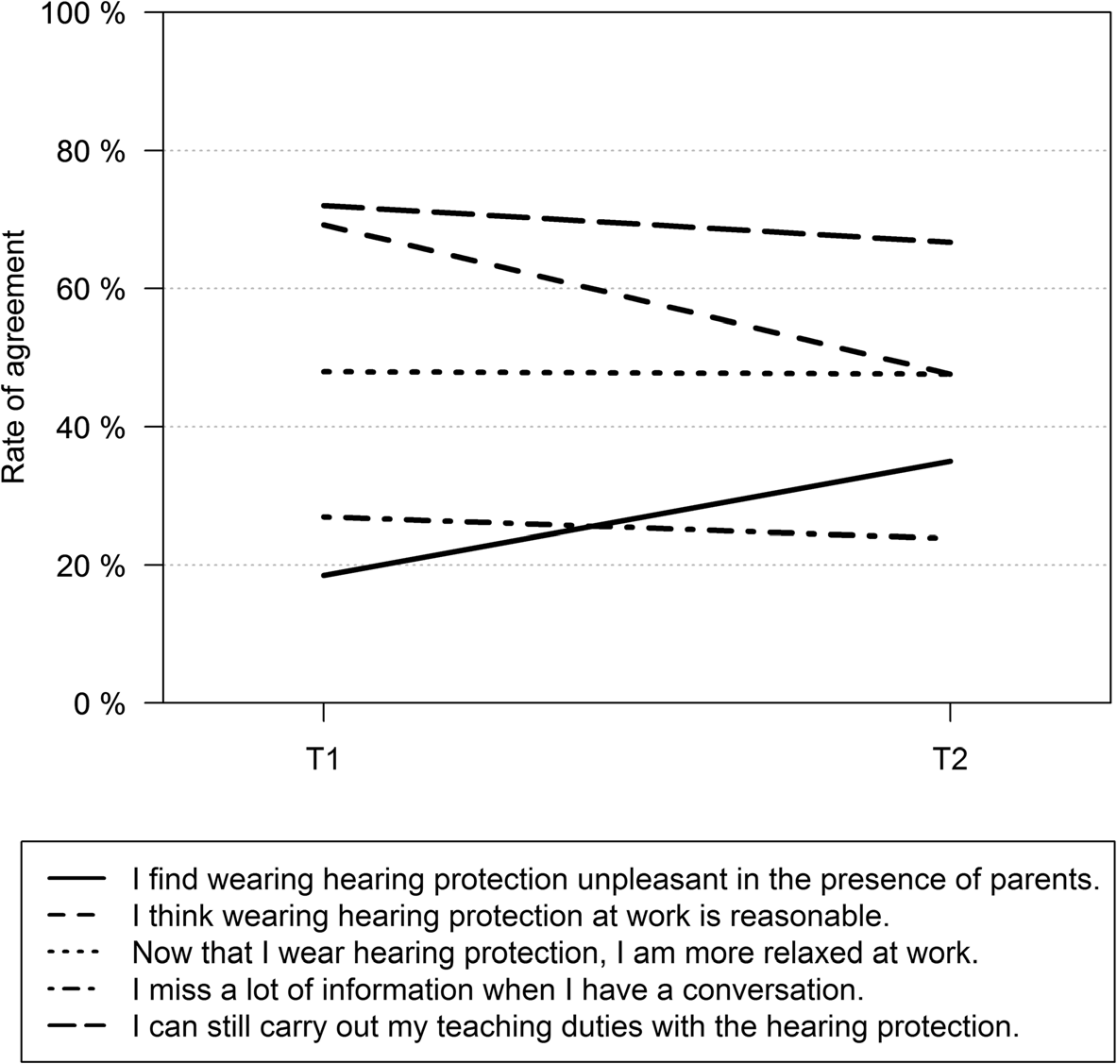
Satisfaction of study participants with the MHPs

Figure 3 shows the information on the satisfaction of the study subjects with wearing MHPs at just over the half of the observation period (T1) and at the end of the period (T2). Satisfaction tended to decrease over time. For example, the fraction of those who found it unpleasant to wear hearing protection in the presence of parents rose from 18 to 35 %. The fraction of those who thought it was reasonable to wear MHPs sank from 69 to 47 %. The fraction of those who experienced relaxation after work remained constant over time (48 %). The fraction of subjects who missed information slightly improved over time - from 27 to 23 %. Almost three quarter of study subjects (72 %) thought that they could fulfil their teaching duties at time point T1. At time point T2 this value decreased to 67 %. None of these changes over time in the dichotomous variables was statistically significant.

Table 5 describes the distribution of study subjects over the 16 different institutions at T0 and T2. The number of subjects at T0 was between one and seven employees per institution. At time point T2, there was no longer any subject from two of the institutions. The number per institution varied between 1 and 5 persons.

**Table 5:** Overview of room acoustic measurements

Institutions		Participants		Mean reverberation time in seconds (T0): **(actual)** (target)	Room acoustic improvements recommended by the room acoustics expert
		T0 (*N*)	T2 (*N*)		
Child day care centres	C_1_	2	2	**0.48** (0.50)	No
	C_2_	3	1	**0.46** (0.40), **0.47** (0.40), **0.73** (0.50)	Yes
	C_3_	3	3	**0.60** (0.50), **0.45** (0.40), **0.60** (0.40)	No
	C_4_	1	0	**0.48** (0.50), **0.60** (0.45), **0.50** (0.40)	No
	C_5_	3	2	**0.38** (0.45)	No
	C_6_	3	2	**0.48** (0.40), **0.56** (0.50)	Yes
	C_7_	3	3	**0.35** (0.40), **0.44** (0.50)	Yes
	C_8_	5	4	**0.64** (0.55), **0.49** (0.40), **0.37** (0.40), **0.58** (0.50)	Yes
	C_9_	7	5	**0.70** (0.55), **0.54** (0.50), **0.61** (0.50)	Yes
	C_10_	2	1	**0.53** (0.40), **0.45** (0.40), **0.89** (0.50)	Yes
	C_11_	4	4	**0.42** (0.50)	No
School partnerships	S_1_	1	1	**0.57** (0.55), **0.66** (0.55)	No
	S_2_	3	2	**0.47** (0.50), **0.60** (0.50), **0.69** (0.50), **0.73** (0.80)	Yes
	S_3_	2	0	**0.85** (0.65), **0.88** (0.65), **0.50** (0.50)	Yes
	S_4_	2	2	**0.64** (0.60), **0.82** (0.60), **0.57** (0.50), **0.97** (0.70)	Yes
	S_5_	1	1	No measurements	No information

Bold: actual, not bold: target

Reverberation times were measured between one and a maximum of four rooms in the different institutions (Table 5). No improvements in the room acoustics were evaluated in 6 institutions, as indicated by the reverberation time measurements and other room characteristics. For the other institutions, improvements in room acoustics were recommended for at least one room. In one institution, no measurement could be performed for organisational reasons. The target values for reverberation times depend on the functions and sizes of the rooms; these values were exceeded in 29/39 measurements (74 %).

## Discussion

When child care workers used MHPs in the present setting, their subjective noise exposure and risk of burnout was not reduced over the period of observation.

It was also observed that most of the subjects did not consider it to be very reasonable to wear MHPs and that this reduced their ability to fulfil their teaching duties over time.

As regards the room acoustics, improvements in room acoustics were recommended for more than half of the institutions on the basis of the measured reverberation times and the structural properties of the rooms.

### Limitations

This study is a scientific investigation of the effect of MHPs. On the other hand, it is also a preventive measure in occupational medicine, which is intended to be available to all employees. In contrast to a classical RCT, this study was performed without a control group or randomisation and without monitoring of other conditions. As a post-hoc analysis the reference group was included to show to what extent the study was performed under changing overall conditions. As the burnout risk was even greater in the reference group, it is clear that there were uncontrolled factors that influenced these outcome variables in both groups. As the threshold for inclusion was low, it can also be assumed that the group was relatively heterogeneous. At the start of the study, the subjects were not subjected to audiometric controls with respect to, for example, hardness of hearing, tinnitus or ear noises, so that the status of their hearing is unknown. Thus, it remains possible that their hearing was heterogeneous. This has the advantage that an occupational preventive measure should be open to as many employees as possible.

A failure of unknown reason in the implementation of the intervention conclusively led to little cumulative wearing time. Consequently it was statistically unlikely to detect any potential dose–response relationship between the wearing time and outcome variables. Qualitative interviews, that could have shed light upon the reasons for the failed implementation of the intervention, were not performed in this pilot study. Because of the size of the sample and the follow-up rate of 73 %, it was hardly possible to demonstrate statistically significant effects; the expected power of 0.80 was not reached in this study, apart from the unforeseen development of the outcome variables. This also applied to the drop-out analysis. This did not allow any conclusion as to whether there was no selection bias, or whether the low number of participants prevented the demonstration of statistically significant variables. Moreover, the non-responders survey (3 of 12 questionnaires returned) did not permit any reliable conclusion about the reasons for non-participation either.

Measurements of sound pressure would have provided an objective estimate of noise exposure, and there are already reference values from German kindergartens. These measurements - including audiometry - had originally been planned for the start of the study. However, they were postponed for organisational reasons and would finally have had to be carried out a long time after the end of the observational period. We therefore eventually decided to dispense with these measurements.

### Intervention

The entry of the wearing times in the diary is intended to be a sort of quality assurance for the intervention. For 6 persons (18 %), there were no entries in the diaries for the wearing times, i.e. it was not known whether these persons had actually worn the MHPs. In accordance with the *intention to treat* principle, these persons were included in the analysis. With the median cumulative wearing time of 34.6 h over 12 months and full employment (224 working days in 2015), this corresponds to a mean daily wearing time of maximally 9 min for half of the participants. As there are no other studies on this type of intervention, there are no reference values and it is difficult to assess whether this is an effective period of time with respect to noise exposure. In the initial workshop, it was emphasised that the participants should wear MHPs at times of peak noise. If time dependent measurements of sound pressure had been performed in the different institutions over the shifts, it might have been possible to relate these to the wearing times. On the other hand, if you consider the statements on satisfaction with MHPs (teaching duties, reasonableness, relaxation effect etc.), it seems more likely that in a substantial number of cases the MHPs were not worn much, because they were disturbing at work.

Comparison with the reference group makes it clear that, at baseline, the intervention group exhibited higher values for both noise exposure and burnout. Especially the difference in subjective noise exposure indicates self-selection of the intervention group. In both groups, burnout risk increased over time. The mean burnout risk and the prevalence in the intervention group (T2: 57.7 and 72.7 %, respectively) appear to be comparatively high. The 2013 COPSOQ database gives a mean reference value of 48 for German child care workers (data in Supporting Information); Buch and Frieling give a burnout prevalence of 30 % [1]. The increase in the burnout risk was smaller in the intervention group, which might indicate that the intervention had a favourable alleviating effect. No factors which might have influenced the intervention effect could be identified in the multivariate analyses. In contrast, it was observed that changes in noise exposure over time were less favourable for subjects who stated in the questionnaire that breaks and possibilities for recovery were missing; thus, the increase in the mean noise exposure values was 5.7 points greater for these persons.

The room acoustic evaluation, including measurements of reverberation time, showed that improvements were possible in 9 of 15 institutions. In some cases, the reverberation times were too high, but in other cases the room acoustics could be improved even though the reverberation times were moderate. Possible problems include wrongly mounted shock absorber elements, missing impact sound insulation, metal doors or too many window surfaces. These assessments make it clear that room acoustics may be suboptimal even when the reverberation times are moderate in accordance with the DIN standard. It was striking - albeit not statistically significant - that employees from institutions where the room acoustics could be improved exhibited higher values of the outcome variables - particularly burnout risk - at both time points. Specific improvements, particularly in these institutions, would therefore benefit the group of employees under the greatest stress. Studies have shown that improvements in room acoustics in schools can reduce reverberation times and, in some cases, also subjective noise exposure [8, 11, 15]. The use of special toy containers can also bring a major reduction in the level of sound pressure [31]. Aside from structural changes, noise exposure can also be reduced at the organisational level. This includes the use of recovery rooms, noise warning lights, light regulation, teaching that increases sensitivity to noise and speech training for child care workers. However, Sjödin et al. have shown that organisational measures are less effective than room acoustic measures, as they require more work [11].

In summary, this study was based on an idea that was initiated by stressed employees and which was implemented as a behavioural preventive measure with scientific support. Due to this frame several characteristics like lack of randomization and control, use of post-hoc reference group, missing compliance in the intervention group and underpowered conditions limit the validity in this study. Further studies should be also designed in a mixed method approach with additional qualitative research. Overall the results suggest that, in this specific setting, wearing MHPs is not an appropriate occupational measure and therefore could not be effectively implemented. In this context it seems that prevention by technical engineering might be more important than the use of personal protective equipment. Qualitative interviews would have been able to identify the reasons for the lack of compliance here more precisely. In contrast to employees in industry, child care workers are exposed to informative noise that has to be heard and which accordingly includes a high level of potential stress. Perhaps the employees’ feelings of responsibility to the children prevented them from wearing the MHPs regularly.

The structural causes of the noise exposure and possibly also burnout risk in this group are presumably inadequate numbers of employees, excessively large groups and, especially, too few trained child care workers [7]. These issues have long been discussed by politicians with expertise in employment and could only be modified at another level. In general, the motivation for this study was typical of the overall conditions for child care workers in Germany, with unfavourable ratios of children to child care workers and lack of expert staff.

## Conclusion

The use of MHPs by child care workers in child day care centres is not an appropriate measure to prevent noise exposure if it is widely employed. For groups of employees with specific problems such as hyperacusis after acute hearing loss further studies are needed. Within the institutions, a careful analysis should be performed of the room acoustics, followed by modification as necessary. In addition, organisational measures e.g. noise education for children should be implemented that have a favourable effect on the initiation and development of noise and its effects on health.

## Abbreviations

BGW: German Social Accident Insurance Institution for the Health and Welfare Services
BMI: Body mass index
COPSOQ: Copenhagen Psychosocial Questionnaire
dB: Decibel
ERI: Effort reward imbalance
KFZA: Short Questionnaire on Work Analysis
MHPs: Moulded hearing protectors
